# Efficient Recycling Blast Furnace Slag by Constructing Ti-Embedded Layered Double Hydroxide as Visible-Light-Driven Photocatalyst

**DOI:** 10.3390/ma15041514

**Published:** 2022-02-17

**Authors:** Ningning Song, Yongfeng Cai, Lingmin Sun, Peng Hu, Qinqin Zhou, Junshu Wu, Jinshu Wang

**Affiliations:** Key Laboratory of Advanced Functional Materials, Education Ministry of China, Faculty of Materials and Manufacture, Beijing University of Technology, Beijing 100124, China; snn@emails.bjut.edu.cn (N.S.); caiyf0127@emails.bjut.edu.cn (Y.C.); 13053593786@163.com (L.S.); zhouqinqin@njut.edu.cn (Q.Z.); junshuwu@bjut.edu.cn (J.W.)

**Keywords:** titanium-containing blast furnace slag, layered double hydroxide, Ti-embedding, visible light response, photocatalysis

## Abstract

In this work, a strategy of heat treatment-precipitation has been developed to recycle Ti-containing metallurgical solid waste by forming Ti-embedded MgAl layered double hydroxide (TMA-LDH). This facile and simple route is featured by the dedicated utilization of the composition of slag with high overall recovery efficiency. Importantly, as-obtained product exhibits visible light response distinctly different from that of pristine MA-LDH ascribed to the Fe doping inherited from initial slag. Its mesoporous nanostructure also provides more microchannels for mass and carrier transfer. As such, excellent photocatalytic activity towards degradation of tetracycline hydrochloride is achieved, and 88% removal could be obtained in 60 min. Furthermore, 44% increase in efficiency than that of Ti-excluded LDH also indicates the synergistically promoting effect of Ti incorporation. Mechanism investigation suggests that Ti incorporation regulates the electronic structure of pristine LDH with more active sites, and favors the formation of radicals with improved oxidative ability for photocatalysis.

## 1. Introduction

Titanium-containing blast furnace slag (Ti-BFS) is a by-product of the iron smelting process as metallurgical solid waste, and mainly composed of perovskite and alumino-silicates of calcium and magnesium, respectively [1]. Because of the large consumption of iron in modern society, the yield of Ti-BFS is quite huge and more than 3 million tons accumulates every year [2]. However, Ti-BFS with low content of Ti (10–25 wt. %) is hard to utilize as raw material directly for Ti manufacture. Concentrating Ti by previously developed strategies, such as acid leaching [3], alkali molten salt calcining [4], carbonization-chlorination [5] and high-temperature enrichment [6], suffers from low efficiency and high cost. Currently, the mostly available route for Ti-BFS utilization is used as a whole for building materials considering its high content of CaO and SiO_2_, but excessive TiO_2_ (>10%) in Ti-BFS would suppress the polymerization degree of building materials and result in a negative effect on its volume stability [7]. As such, most of the Ti-BFS is disposed directly without further treatment, which causes the waste of Ti-containing secondary resource and poses a risk to the environment; thus, developing facile and efficient approaches to recycle Ti-BFS has become a critical issue needing to be seriously considered.

Layered double hydroxide (LDH) is a type of two-dimensional inorganic compound, in which bivalent (such as Mg^2+^, Zn^2+^, Ni^2+^) and trivalent metallic cations (such as Al^3+^, Fe^3+^, In^3+^) construct the layered framework, while exchangeable anions occupy the interlayer region for charge compensation [8]. Owing to its unique structure and synergistic effect of bimetals, LDH is endowed with exceptional properties, and has been widely applied in adsorbents [9,10], catalysts [11,12], energy storage [13,14], drug delivery [15,16], etc. Specially, LDH possesses abundant hydroxyl groups, which provides potential active hydroxyl radicals to break the ring structure of organic compounds (such as toluene, TC) excited by light irradiation. In addition, the typically sheet-like structure also provides high surface to volume ratio, and enriches the active sites on the surface to further promote the photocatalytic reaction. As such, LDH exhibits great potential as efficient photocatalyst for organic pollutant degradation. Up to now, various routes have been developed for LDH preparation, such as co-precipitation, hydrothermal, anion exchange and sol-gel methods [17,18,19,20]. Among these methods, alkali-precipitation has attracted much attention due to its simple and low-cost features, and brings LDHs closer to practical applications. Considering the existence of bivalent Mg and trivalent Al in Ti-BFS with high content, it is reasonable to expect that Ti-BFS can be served as potential raw material for MgAl-LDH preparation, which not only achieves the valuable utilization of Ti-BFS, but also decreases the cost of LDH. More recently, Xiao et al. explored the synthesis of CaFeAl-LDH by using calcium carbide slag and red mud composites as raw material, and the as-obtained product exhibits high ability for phosphorus adsorption [21]. However, the thermal treatment of calcium carbide possibly causes the formation of harmful carbon monoxide or carbon oxide gas, and detailed recovery efficiency has not been investigated in their work. As such, further work still needs to be conducted to properly address the facile and mild synthesis of LDH from metallurgical waste with acceptable extraction efficiency, and provide more promising recycling strategies for Ti-BFS.

In this work, a heat treatment-precipitation strategy was developed to recycle Ti-BFS efficiently by formation of products including the Si-Ca compound and Ti-embedded MgAl-LDH, respectively. The deliberate utilization of the slag mixture emphasizes the overall recovery efficiency of almost over 90%. Furthermore, the photocatalytic activity of as-obtained MgAl-LDH was evaluated by removal of tetracycline under visible light irradiation, and superior performance was identified by 92% degradation of initial concentration. In addition, enhanced light absorption and synergistic effect of Ti that contributed to the promoted organic pollutant removal were also investigated in detail.

## 2. Materials and Methods

### 2.1. Materials

The Ti-BFS was obtained from Pan Steel Group in Sichuan, China, and the main composition is given in Table 1. All the other chemicals are of analytical grade and used as received without further purification.

### 2.2. Recycling Process of Ti-BFS

**Figure 1:**
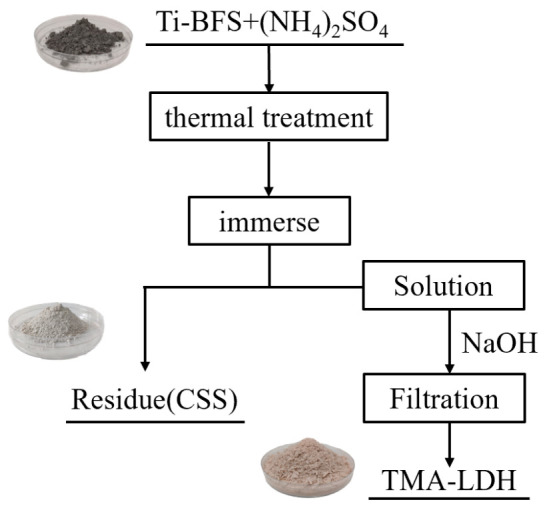
Schematic illustration of the recycling process of Ti-BFS.

The schematic illustration of the recycling process of Ti-BFS is presented in Figure 1. Firstly, Ti-BFS was grinded by ball milling and sieved with 80 mesh screen, and 5 g fine Ti-BFS was mixed with 40 g ammonium sulfate followed by thermal treatment at 450 °C for 1 h. After reaction, the product was immersed into 160 mL water at 80 °C for 2 h, and the insoluble residue was separated by centrifugation and named as CSS. Then the pH of obtained supernatant was adjusted to 10 using 2 mol/L NaOH solution, and aged at 70 °C for 4 h to obtain a suspension. Finally, Ti-embedded hydrotalcite product was collected by centrifugation and washed with deionized water and ethanol, respectively, and further dried at 60 °C in an oven. As obtained product was named as TMA-LDH. The optical images of the samples are shown in the inset pictures of Figure 1.

The recovery efficiency was calculated according to the overall weight and contents of corresponding elements in initial slag, obtained CSS and TMA-LDH, respectively. Based on ICP analysis, the contents of component elements in different products could be estimated, and then the accurate weights were calculated by separating the overall weight according to their relative proportion. Finally, the recovery efficiency of different elements could be determined by their total amount in CSS and LDH compared to the values in initial slag.

To investigate the influence of Ti incorporation on the structure and property of obtained layered double hydroxide, a controlled experiment was conducted to exclude Ti in hydrotalcite by adjusting the pH of supernatant to 4, which results in the formation of TiOSO_4_ precipitation. After filtration, the hydrotalcite without Ti was obtained by further adjusting the pH of supernatant to 10, and as-obtained product was named as MA-LDH.

### 2.3. Characterizations

The phases of obtained products were investigated by powder X-ray diffraction patterns (XRD) on a Bruker D8 Advance diffractometer using Cu-Kα radiation. The elemental composition was determined using inductively coupled plasma spectrometer (ICP, Optima 8300). Scanning electron microscopy (SEM, Hitachi SU8020) and transmission electron micrograph (TEM, FEI Talos F200X-G2) were adopted to investigate the morphologies of samples. The ASAP 2020 volumetric adsorption analyzer was used to measure the nitrogen adsorption-desorption isotherms at 77 K to analyze the specific surface area and average pore volume of the samples. Ultraviolet-visible diffuse reflectance spectroscopy was collected on Shimadzu UV2550 spectrophotometer. The X-ray photoelectron spectroscopy (XPS) spectrum was obtained by an ESCALAB250 X-ray photoelectron spectrometer to reveal the element composition and chemical state of the samples. All XPS peaks of the elements were calibrated against the C 1s line fixed at 284.6 eV as a reference. The ^27^Al high-resolution NMR spectra were obtained at 750 MHz principal field on a Bruker AVANCE III equipped with high-speed MAS probe with 20 mg samples. Electron paramagnetic resonance spectroscopy (EPR) spectra were used to detect the photo-excited active species during photocatalytic reaction on a Bruker EMX spectrometer with 400 mg/L solution.

### 2.4. Photocatalytic Activity Measurement

Photocatalytic activity of the samples was characterized by degradation of tetracycline hydrochloride (TC) under visible light irradiation. A Xe lamp (400 W, Perfect Light) with a cut-off filter of 420 nm was used as light source. In a typical photocatalytic process, 5–25 mg of obtained LDH and 50 mL of 5–100 mg/L TC solution were mixed for photocatalytic reaction, and the suspension was firstly stirred in the dark for 30 min to achieve nearly full adsorption-desorption equilibrium. At specific light illumination intervals, 3 mL of the reaction solution was taken and centrifuged to remove the catalyst sample and analyzed by UV–vis absorption spectroscopy. The residual TC concentration of filtrate was tested according to its absorbance at a wavelength of 375 nm. After one cycle of test, the catalyst was collected by centrifugation, and washed with water and ethanol several times. Then, the catalyst was dried in an oven at 60 °C for 24 h for the next test. Three cycles of tests were conducted to investigate the stability of obtained catalyst.

## 3. Results and Discussion

**Figure 2:**
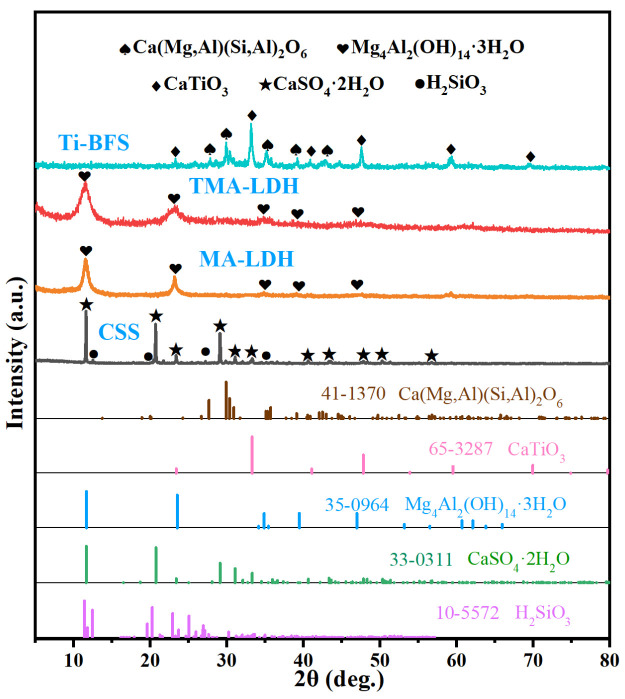
XRD patterns of Ti-BFS and obtained various products.

In order to efficiently recover Ti-BFS, ammonium sulfate was firstly adopted to react with metal cations including Ti, Ca, Mg and Al in Ti-BFS, and corresponding sulfates generated during the thermal treatment process, which facilitates the elemental separation in the following immersing process considering their different solubility in water. Insoluble CaSO_4_ and unreacted SiO_2_ entered solid residue, and can be used as raw materials for cement. Soluble Ti, Mg and Al cations were further precipitated by adjusting the pH of the solution to alkaline state, which results in the formation of Ti embedded MgAl-LDH. The reaction mechanism could be verified by phase evolution of slag during recycling process as illustrated in Figure 2. Compared to the dominated perovskite phase and alumino-silicates of calcium and magnesium phases in pristine Ti-BFS, as-obtained CSS was mainly composed of calcium dihydrate and metasilicic acid, respectively. While the exclusive phase of hydrotalcite-like MgAl-LDH was observed in precipitated TMA-LDH and MA-LDH products, illustrating the formation of layered double hydroxide. In addition, the absence of Ti-related phases in TMA-LDH was possibly ascribed to its efficient incorporation into the framework of LDHs. This feasible alkali precipitation has also been widely adopted to synthesize pristine and doped LDHs in previous work [22,23,24,25].

Furthermore, composition analysis gives quantitative information of element distribution in the obtained products. As illustrated in Table 2, Si and Ca in CSS were identified with weight ratio higher than 90%, indicating good separation and enrichment of Si and Ca during calcination and the immersion process. While the left elements were mostly preserved in LDH, and the existence of Ti in TMA-LDH was also confirmed by composition analysis. It should be noted that sharp decrease of Ti content in obtained MA-LDH could also be identified by elemental analysis as shown in Table S1. In addition, the general weight ratios of main elements converted in CSS and TMA-LDH are listed in Table S2. As indicated, the overall recovery efficiency beyond 90% could be achieved for Si, Ti, Al and Mg, respectively. Furthermore, above results indicate the feasibility of here developed method as a promising strategy for Ti-BFS recycling with high efficiency and additional value.

**Figure 3:**
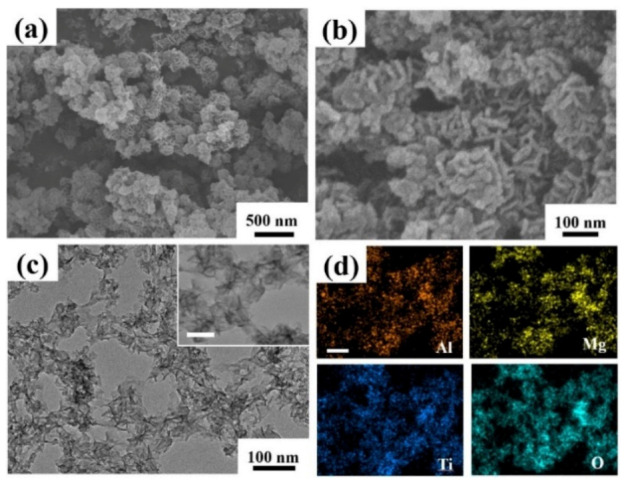
(**a**) Low and (**b**) high magnified SEM images, (**c**) TEM images and (**d**) elemental mapping of obtained TMA-LDH. The scale bars of inserted images in c and d are 50 nm.

The morphology of synthesized TMA-LDH was investigated by electron microscope observation. As indicated in Figure 3a, flower-like particles with size of about 100 nm dominated in the obtained product, and the magnified SEM image in Figure 3b further reveals that the particle was assembled by loosely aggregated nanosheets as building blocks. The detailed structure was also identified by TEM in Figure 3c, and it was observed that assembled nanosheets were connected to each other by the formation of a net-like structure. Enlarged TEM images further illustrate the thinness of the nanosheet according to the brightness contrast between the sample and the substrate. Furthermore, EDS mapping was adopted to visually describe the elemental distribution of LDH, and Al, Mg, Ti and O were well distributed in the product. Considering the uniform morphology and homogeneous elemental distribution, it is suggested that Ti is incorporated into the framework of layered double hydroxide. In addition, the morphology of MA-LDH was also investigated as shown in Figure S1. As indicated, sheet-like nanostructures with less aggregation were observed, which illustrates the shape evolution induced by sharply decreased Ti content.

**Figure 4:**
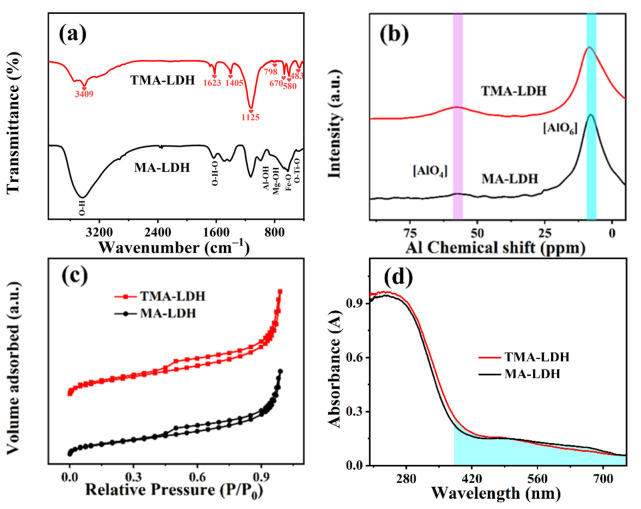
(**a**) FT-IR spectra, (**b**) NMR spectroscopy, (**c**) nitrogen adsorption-desorption isotherm and (**d**) UV-vis absorption spectra of synthesized brucite products.

The structure and property of obtained brucite products were further investigated as shown in Figure 4. FT-IR spectra were firstly adopted to identify the functional groups of synthesized layered double hydroxides as shown in Figure 4a, and the similar functional groups were presented both in MA-LDH and TMA-LDH, respectively. In the case of TMA-LDH, typically stretching vibration of the hydroxyl groups is observed at the band of about 3409 cm^−1^, which is distinct from the vibration of H_2_O molecules at 1629 cm^−1^ [26]. In addition, the peaks at 798 cm^−1^ and 670 cm^−1^ are ascribed to the typical vibrations of Al-OH and Mg-OH bonds in MgAl LDH, respectively [27]. The bonding structure of Ti was recognized as an O-Ti-O bond with the vibration peak at 483 cm^−1^ [28]. Furthermore, a fresh generated peak at 580 cm^−1^ was recognized and attributed to the Fe-O vibration [29], and this result illustrates the existence of Fe in as-obtained product, which is possibly originated from the residual Fe in pristine Ti-BFS.

Layered structure of LDH is featured by the alternatively arranged metals coordinated to six hydroxyl groups in an octahedral geometry, thus six-coordinated (octahedral) AlO_6_ is the typical structure unit of MgAl LDH [30]. This could be verified well by the strong signal of AlO_6_ resonance in NMR spectroscopy shown in Figure 4b. In addition, a weak peak ascribed to four-coordinated AlO_4_ was also observed, suggesting the crystallographic change of aluminum in octahedral positions, and possibly attributed to the structural distortion by Ti containing within the octahedral TiO_6_ configuration [31]. Importantly, decreased AlO_6_ signal and increased AlO_4_ signal in TMA-LDH also illustrates the stronger structural distortion due to its rather high Ti content than that of MA-LDH.

The specific surface area of obtained LDHs was investigated by nitrogen adsorption-desorption isotherm. Type IV isotherms are well presented in Figure 4c, indicating the mesoporous structure of the two products. The specific surface area was further determined to be about 135.76 m^2^/g and 188.44 m^2^/g, together with similar pore sizes of 3.8 nm for TMA-LDH and MA-LDH (Figure S2), respectively. This result suggests the negligible effect of Ti content on the specific surface area of the obtained products. Notably, high surface area with porous structure provides more microchannels for mass transport and active sites for catalytic reaction, and in turn promotes the photocatalytic performance due to the enhanced kinetic nature. Figure 4d gives the UV-vis absorption spectra of TMA-LDH and MA-LDH, and similar curves were identified. Impressively, high absorption shoulder with red-shift absorption edge was observed for two samples, suggesting their enhanced light response of visible light absorption. According to the UV-vis curves, the bandgap energy could be calculated by the Tauc plots shown in Figure S4, and the values are 2.71 eV and 2.79 eV for TMA-LDH and MA-LDH, respectively. It is worth noting that pristine MgAl-LDH can only absorb ultraviolet light [8], which suppresses the efficient utilization of solar energy by more than 50% energy in the visible light zone. Thus, the enhanced ability of light absorption illustrates more available protons excited by light, and is favorable to the following redox reaction. Considering the existence of Fe in obtained LDH and initial slag (Figure S3), it is reasonably speculated that the enhanced visible light absorption is originated from the Fe doping [32].

**Figure 5:**
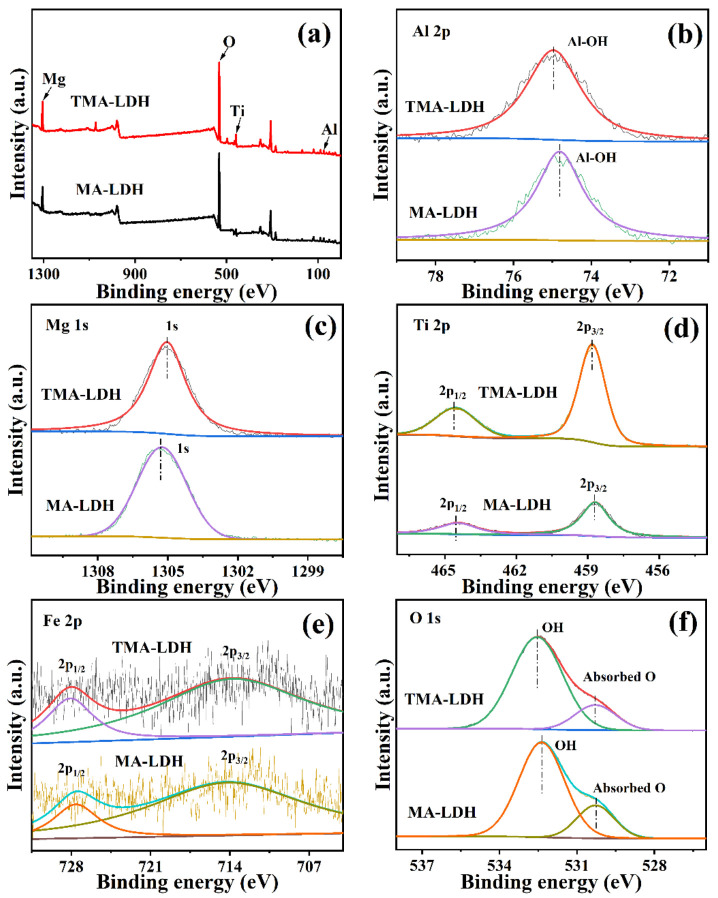
XPS spectra of obtained TMA-LDH and MA-LDH: (**a**)survey, (**b**) Al 2p, (**c**) Mg 1s, (**d**) Ti 2p, (**e**) Fe 2p and (**f**) O 1s.

The chemical composition and valence state on the near surface of as-synthesized LDHs were investigated by XPS analysis. All XPS peaks of the composition elements were calibrated against the C 1s line fixed at 284.6 eV (XPS spectra of C are given in Figure S5). According to the overview measurement in Figure 5a, Al, Mg, Ti and O could be detected both in TMA-LDH and MA-LDH, respectively. Figure 5b presents the high-resolution spectra of Al 2p, and the peak located at 74.8 eV could be attributed to the Al–OH structure in the brucite-like layer of MA-LDH [33]. Apparently, the band energy of Al 2p was increased to 75.0 eV for TMA-LDH, indicating the enhanced electron density around Al sites [34,35]. In addition, the binding energy of Mg 1s in Figure 5c reveals its chemical states of oxide/hydroxide at 1035.3 eV in MA-LDH [36]. The decreased binding energy of 0.3 eV for TMA-LDH suggests the possible electron transfer from Mg to Al after Ti incorporation, and results in redistribution of spatial electron density around Mg and Al sites, respectively. Because the intrinsic electronic structure directly determines the surface chemical behavior that is highly related to the catalytic properties; adjustable photocatalytic performance is expected by modulating MA-LDH through Ti incorporation.

Ti 2p spectra of TMA-LDH and MA-LDH exhibit two separate peaks assigned to the Ti^4+^ 2p_3/2_ and Ti^4+^ 2p_1/2_ [37], respectively (Figure 5d), but the peak intensity is quite different for two samples. The rather strong intensity of TMA-LDH indicates its high content of Ti, which is well in accordance with the composition analysis. In addition, the increased binding energy of Ti^4+^ 2p_3/2_ and Ti^4+^ 2p_1/2_ in TMA-LDH could also be observed, illustrating the effective Ti embedding in the framework of LDH. XPS fitting of Fe 2p (Figure 5e) is to provide a qualitative illustration of iron on the near surface of LDHs. Two peaks with the binding energy of 713.60 eV and 727.00 eV could be recognized and well assigned to Fe 2p_3/2_ and Fe 2p_1/2_, respectively [38,39]. The detailed Fe content is determined to be about 0.5 wt. % by ICP analysis. Figure 5f gives the core level spectrum of O 1s, and two fitted peaks with binding energy of about 532.3 eV and 530.2 eV could be observed, which are ascribed to the hydroxyl groups and adsorbed oxygen species [40], respectively. It should be noted that Ti incorporation also increases the electron density around the O site, which is clearly indicated by the positive shift of its energy band, possibly due to the crystallographic change as illustrated in Figure 5b.

**Figure 6:**
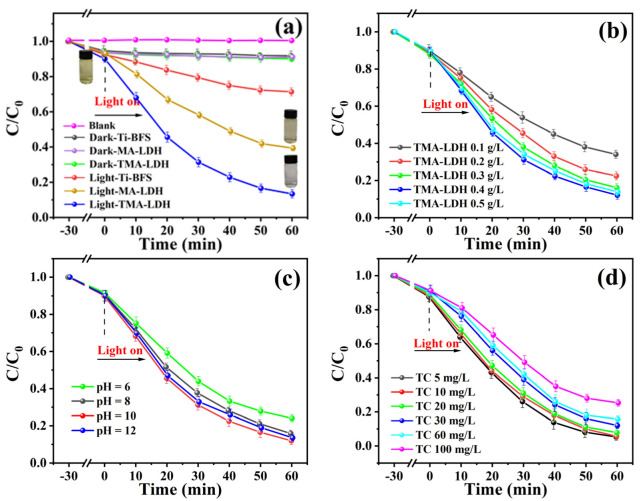

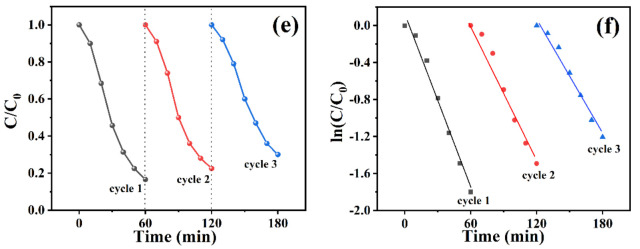
Photocatalytic performance of as-synthesized catalysts towards degradation of TC under visible light irradiation: (**a**) photo-degradation efficiency as function of reaction time; effect of (**b**) catalyst dosage, (**c**) initial pH value and (**d**) initial TC concentration on the photo-degradation efficiency of TC; (**e**) stability of TMA-LDH for TC degradation and (**f**) reaction kinetics analysis of three cycles of photocatalysis reaction.

Considering the high surface area and enhanced light absorption ability, as-obtained LDH is expected to present high performance in photocatalysis. Here, the degradation behaviors of TC over different catalysts were investigated under visible light (λ > 420 nm), and presented in Figure 6a. As indicated, Ti-BFS, MA-LDH and TMA-LDH achieve adsorption-desorption equilibrium after 90 min stirring in the dark, and the adsorption efficiency is determined to be about 8.11%, 8.85% and 9.31%, respectively. Before photocatalytic testing, 30 min stirring was maintained in the dark to achieve nearly full adsorption-desorption equilibrium, and the adsorption efficiencies are about 5.40%, 6.11% and 6.42%, respectively. According to the degradation curve, Ti-BFS shows rather low photocatalytic activity, and only 30% TC was degraded after reaction for 60 min. With the introduction of MA-LDH as photocatalyst, the degradation efficiency was significantly enhanced to 61%. Importantly, rather low TC concentration of 12% was further obtained when adopting TMA-LDH as catalyst. The degradation efficiency is about 2.9 times higher than that of Ti-BFS, and 44% increase than that of MA-LDH, which illustrates the remarkably promoting effect well of Ti on the photocatalytic activity of obtained LDH. It should be noted that TC photolysis presents negligible contribution on the degradation efficiency of photocatalysts.

Figure 6b gives the influence of catalyst dosage on the degradation efficiency of TC. Upon increasing the amount of LDH, the photocatalytic activity was firstly enhanced, and reached the highest value at 0.4 g/L. When the usage of catalyst exceeded 0.4 g/L, the degradation efficiency was further decreased. The reason is that excessive catalyst would shield the light transmittance in the solution, and insufficient photon flux was received for the catalysts far away from the light source, which hinders the catalytic reaction and suppresses the degradation efficiency. Similar results were also observed for photocatalytic tests conducted under different pH and TC concentrations, as illustrated in Figure 6c,d.

To assess the stability and recyclability of TMA-LDH catalyst, three cycles of photocatalysis tests were conducted and the results are displayed in Figure 6e. Compared to 88% degradation of TC during the first cycle, 70% removal of initial TC could be obtained during the third cycle, indicating 18% decay of photocatalytic capacity after three cycles. Furthermore, the kinetic feature of photocatalyst over three cycles of reaction was also investigated as shown in Figure 6f. It is obvious that the photo-degradation rates can be well fitted to pseudo-first-order kinetics according to the equation of ln(C/C_0_) = k_app_t, where k_app_ represents the rate constant and t is the reaction time. Thus, k_app_ of three cycles of reaction was identified to be about 0.030, 0.024 and 0.021, respectively. The well-preserved k_app_ with high values not only indicates the favorable kinetic feature, but also suggests the stable reaction rates during the cycled photocatalytic process. Thus, the feasibility of as-synthesized catalyst as potential candidate for practical applications was well illustrated.

**Figure 7:**
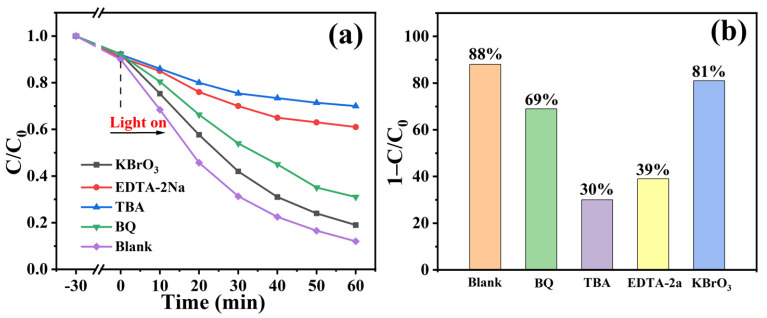

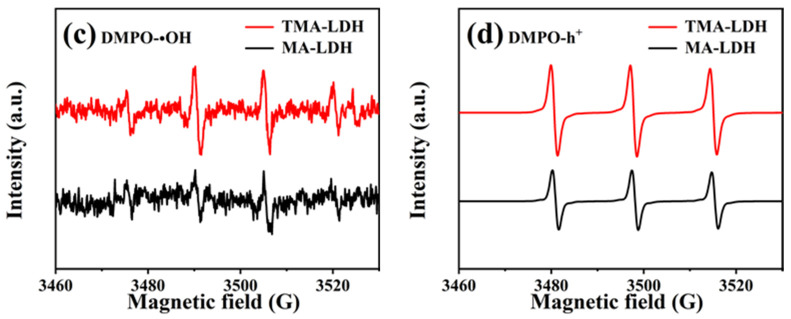
(**a**) Photo-degradation of TC and (**b**) comparison of degradation efficiency of TMA-LDH with presence of different radical scavengers, (**c**) EPR spectra of DMPO-·OH and (**d**) DMPO-h^+^ of obtained TMA-LDH and MA-LDHs.

To obtain intrinsic understanding of the photocatalytic mechanism, radical capture experiments were designed to identify the active species during the reaction process. Scavengers including tertiary butanol (TBA), 1,4-benzoquinone (BQ), potassium bromate (KBrO_3_) and ethylene diamine tetra acetic acid disodium salt (EDTA-2Na) were adopted to quench the hydroxyl radicals (·OH), superoxide radicals (O_2_^−^), photogenerated electrons (e^−^) and holes (h^+^), respectively. As indicated in Figure 7a, b, the degradation efficiency of TC was remarkably suppressed by adding TBA and EDTA-2Na, and the values were determined to be 30% and 39%, respectively. While 69% decrease in photocatalytic efficiency was observed with existence of BQ. However, addition of KBrO_3_ exhibited a slight effect on the degradation rate of TC. Thus, ·OH and h^+^ excited on the valence band of TMA-LDH were identified as the predominant active species rather than ·O_2_^−^ and e^−^ on the conduction band, and oxidation reaction was recognized as the main mechanism for the photocatalytic removal of TC over TMA-LDH catalyst.

ESR spectra were also adopted to investigate the promoting mechanism of Ti incorporation on the photocatalytic activity of obtained product. As illustrated in Figure 7c, d, the signals of ·OH and h^+^ under light irradiation were detected for two LDHs, which is well consistent with the results of the trapping experiments. Furthermore, relatively stronger signals both for ·OH and h^+^ could be observed for MA-LDH, and suggests the generation of more active species under visible light irradiation. As such, it is reasonably speculated that Ti incorporation promotes the formation of active species during photocatalytic reaction, and is responsible for the greatly enhanced performance of LDH.

Heteroatom doping is an efficient strategy to regulate the electronic structure of semiconductors [41]. By partially destroying the periodicity of the crystal lattice, locally re-distributed electronic structure was achieved. This modification effectively changes the chemical behaviors of the reaction intermediates, and in turn enhances the catalytic ability of catalysts [34]. Previously theoretical and experimental work also reveal the feasibility of heteroatom doping on promoting the catalytic activity of LDHs [42]. Specially, Zhang et al. reported that high-valence state metal (Ta) caused the lattice expansion of LDH, and resulted in the modified e_g_ orbital of Ta by directional charge transfer. Thus, Ta could serve as freshly generated active sites with preferred adsorption ability for intermediate species, and further improved the catalytic activity due to the enhanced reaction kinetics [43]. Furthermore, Kang et al. illustrated that the bonding nature for a mixed oxide structure of LDHs could be well adjusted by Ti embedding, and resulted Ni/Ti LDH exhibited high photocatalytic activity towards water oxidation [44]. In our work, Ti incorporation exhibited negligible effect on the surface area and light absorption, but the enhanced oxidation ability was achieved and ascribed to the increased active species of ·OH and h^+^ excited on the valence. As such, the modulated electronic structure is suggested to be the intrinsic mechanism for the improved photocatalytic performance, which is also confirmed by the structural investigations, such as XPS and NMR.

**Figure 8:**
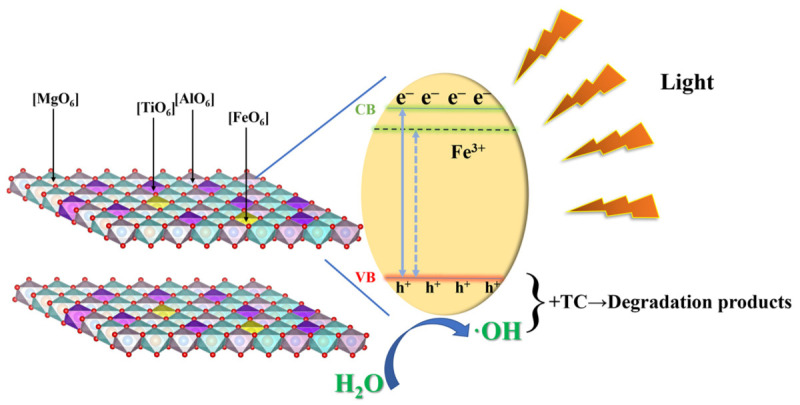
The mechanism of photo-degradation of TC by TMA-LDH under visible light.

The degradation pathway of TC during photocatalysis has been investigated by many works. Firstly, the functional groups with high energy (such as amine groups and phenol groups) were interacted with active species and detached from TC molecules [45]. Then the hydrolyzed TC further lost its functional groups as the reaction proceeds, together with broken cyclic hydrocarbon structure [46]. After a series of redox reactions, the intermediates were mineralized and generated final products of CO_2_ and H_2_O [47]. Based on the above discussion, it is reasonable to summarize the mechanism of increased photocatalytic activity of TMA-LDH catalyst, and the schematic illustration is presented in Figure 8. Under irradiation with visible light, photo-induced holes can be excited to the valence band of catalyst and further oxidizes OH^−^ to ·OH radicals, then the generated h^+^ and ·OH degraded TC into other small molecules. Due to the modulated electronic structure of MA-LDH by Ti incorporation, more active sites on the surface were exposed and favored the formation of radicals, which promotes the catalytic reaction with enhanced kinetic feature. In addition, increased ability of visible light absorption provided more photo-excited carriers that participated in the redox reactions, and mesoporous nanostructure also promised more microchannels for mass and carrier transfer, which all contributes to the excellent photocatalytic activity for TC removal.

## 4. Conclusions

In summary, we have demonstrated a heat treatment-precipitation strategy to efficient recycle Ti-containing blast furnace slag as visible-light-driven photocatalyst for TC removal. As indicated, Ti-embedded MgAl layered double hydroxide and Si-Ca-contained compound are obtained as products, which accounts for the overall recovery rate almost higher than 90%. This recycling method also endows synthesized product with mesoporous structure and increased light response ability in visible range, and benefits the photocatalytic reaction. More importantly, controlled experiments suggest that Ti incorporation modifies the electronic structure of pristine LDH, which favors the formation of more available radicals and is responsible for the remarkably promoted photocatalytic activity. As a result, 88% degradation of initial concentration of TC could be achieved when adopting synthesized TMA-LDH as catalyst. The degradation efficiency is about 2.9 times higher than that of Ti-BFS, and a 44% increase compared to that of MA-LDH, respectively. Accordingly, as-proposed recycling strategy with high utilization efficiency addresses it as a feasible approach to the recovery of other slags with excellent properties for various applications.

## Figures and Tables

**Table 1:** The main composition of Ti-BFS.

Composition	CaO	SiO_2_	TiO_2_	MgO	Al_2_O_3_
wt. %	28.91	26.16	21.45	9.05	14.43

**Table 2:** Element distribution in different products and corresponding recovery efficiency.

Compositional Element	CSS (wt. %)	TMA-LDH (wt. %)	Recovery Efficiency (wt. %)
Ca	55.67	5.71	88.11
Si	37.59	1.74	96.00
Ti	5.30	52.19	95.26
Al	0.05	17.83	97.14
Mg	1.39	22.53	91.87

## Data Availability

Not applicable.

## Supplementary Materials

The following supporting information can be downloaded at: https://www.mdpi.com/article/10.3390/ma15041514/s1, Figure S1: (a) Low and (b) high magnified SEM images, (c) TEM images and (d) elemental mapping of obtained MA-LDH; Figure S2: Pore size distribution of obtained brucite products; Figure S3: XPS spectra of Fe 2p in Ti-BFS; Figure S4: Band gap energy of TMA-LDH and MA-LDH; Figure S5: XPS spectra of the carbon component; Table S1: Main composition of MA-LDH; Table S2: General weight ratios that converted CSS and TMA-LDH.
