# Supplementary material for: Efficient Recycling Blast Furnace Slag by Constructing Ti-Embedded Layered Double Hydroxide as Visible-Light-Driven Photocatalyst
Source: Materials (Basel). 2022 Feb 17;15(4):1514. doi: 10.3390/ma15041514 (PMC8877268; doi:10.3390/ma15041514)
Supplement: Supplementary file 1 [file materials-15-01514-s001.zip › materials-1584671-supplementary.pdf]

Supplementary Materials

Efficient Recycling Blast Furnace Slag by Constructing Ti-Embedded Layered Double Hydroxide as Visible-Light-Driven Photocatalyst

Ningning Song, Yongfeng Cai, Lingmin Sun, Peng Hu *, Qinqin Zhou, Junshu Wu and Jinshu Wang *

Key Laboratory of Advanced Functional Materials, Education Ministry of China, Faculty of Materials and Manufacture, Beijing University of Technology, Beijing 100124, China; snn@emails.bjut.edu.cn (N.S.); caiyf0127@emails.bjut.edu.cn (Y.C.); 13053593786@163.com (L.S.); zhouqinqin@njut.edu.cn (Q.Z.); junshuwu@bjut.edu.cn (J.W.)

* Correspondence: pengh@bjut.edu.cn (P.H.); wangjsh@bjut.edu.cn (J.W.)

Table S1. The main composition of MA-LDH

Composition	CaO	SiO_2	TiO_2	MgO	Al_2O_3
wt. %	5.77	2.01	8.75	30.14	53.33

Table S2. General weight ratios that converted in CSS and TMA-LDH

Elements	TMA-LDH (wt. %)	CSS (wt. %)
Ca	6.00	82.11
Si	2.38	93.62
Ti	82.69	12.57
Al	92.96	4.28
Mg	84.09	7.78

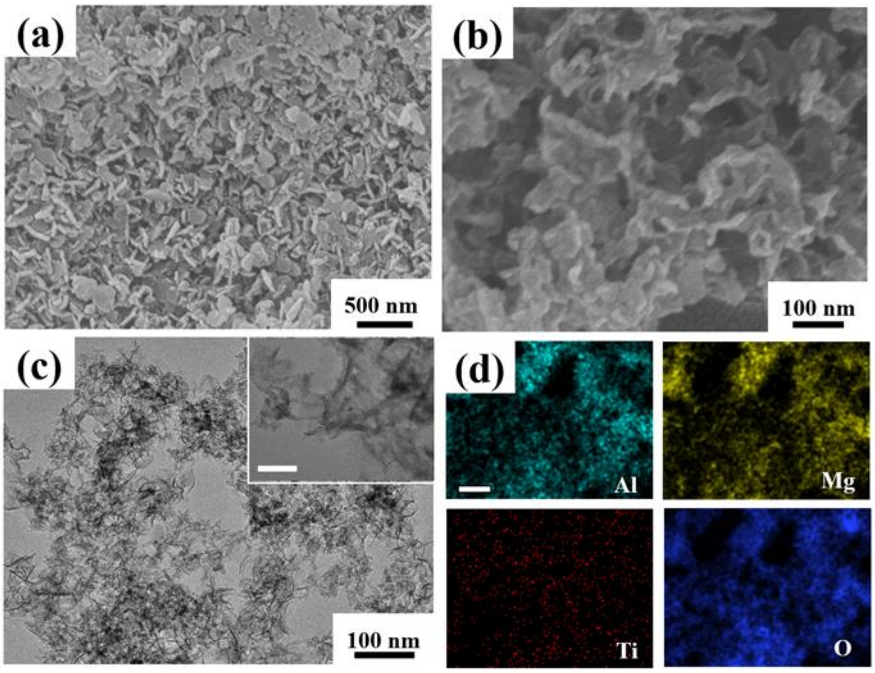

Figure S1. (a) low and (b) high magnified SEM images, (c) TEM images and (d) elemental mapping of obtained MA-LDH. The scale bar of image inserted Figure c is 50 nm.

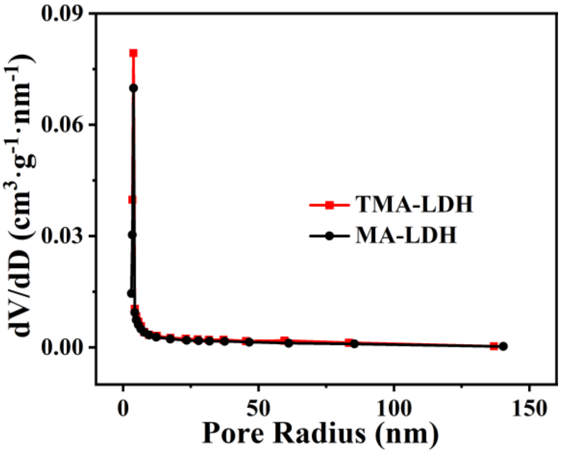

Figure S2. Pore size distribution of obtained brucite products.

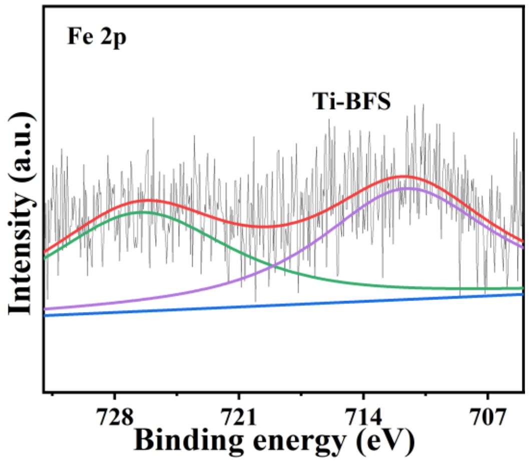

Figure S3. XPS spectra of Fe 2p in Ti-BFS.

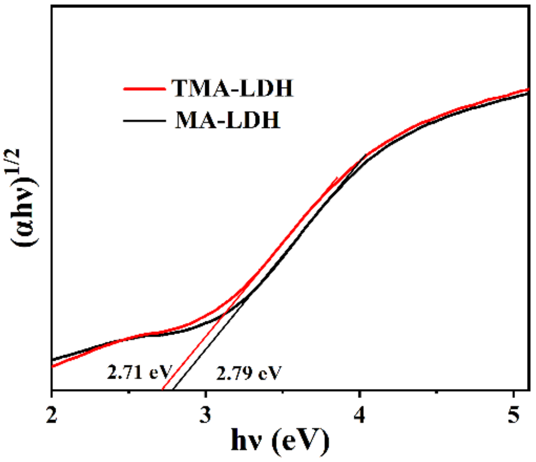

Figure S4. Band gap energy of TMA-LDH and MA-LDH.

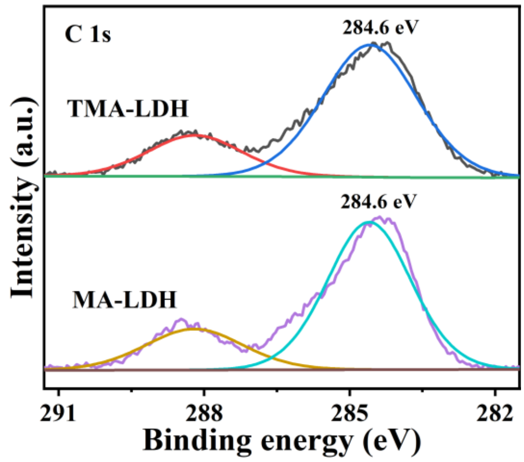

Figure S5. The XPS spectra of the carbon component.
